# National Convention for Revising the Pharmacopœia of the United States

**Published:** 1860-07

**Authors:** 


THE NATIONAL CONVENTION FOR REVISING THE PHARMACOPŒIA OF THE UNITED STATES, held its fifth decennial meeting in Washington City, on the second of May, and was in session two days. The following gentlemen were elected permanent officers of the Association:—*President*, Dr. George B. Wood, of Philadelphia; *Vice-Presidents*, Dr. Jacob Bigelow, of Boston, and Dr. Edward Warren, of North Carolina; *Secretary*, Dr. Thos. Miller, of Washington; *Assistant Secretary*, Dr. John C. Riley, of Washington. The delegates of several medical societies and colleges of pharmacy presented reports toward a revision of the National Pharmacopœia, which were referred to a committee, with directions to report a plan for its revision and publication; the committee consisted of Drs. Bache, Miller, and Russell, and Messrs. Sharp and Parrish. From this committee Dr. Bache brought in a report comprising a series of resolutions, one of which was that there should be a committee of nine (Dr. Wood to be one) to revise and publish the Pharmacopœia; also, that three be a quorum, and that the place of meeting be in Philadelphia. This report was accepted, and the following committee appointed:—Dr. Franklin Bache, Dr. E. R. Squibb, Dr. T. C. Carney, Dr. G. B. Wood, Dr. H. T. Cumming, Dr. W. Procter, Mr. Jos. Carson, Mr. W. T. Thompson, and Mr. A. B. Taylor. To the Committee on Revision and Publication it was recommended that in the index to the Pharmacopœia the syllables of both Latin and English names be so divided and accented that the index may also serve as a pronouncing vocabulary to the Materia Medica. Various other subjects were discussed by the Convention, including that of the uniformity of weights and measures; but our limited space prevents us giving a fuller abstract of its proceedings.

